# Evaluation of Decalcification Techniques for Rat Femurs Using HE and Immunohistochemical Staining

**DOI:** 10.1155/2017/9050754

**Published:** 2017-01-26

**Authors:** Haixia Liu, Ruyuan Zhu, Chenyue Liu, Rufeng Ma, Lili Wang, Beibei Chen, Lin Li, Jianzhao Niu, Dandan Zhao, Fangfang Mo, Min Fu, Dieter Brömme, Dongwei Zhang, Sihua Gao

**Affiliations:** ^1^Preclinical Medicine School, Beijing University of Chinese Medicine, Beijing 100029, China; ^2^Chinese Material Medica School, Beijing University of Chinese Medicine, Beijing 100029, China; ^3^Diabetes Research Center, Beijing University of Chinese Medicine, Beijing 100029, China; ^4^The Research Institute of McGill University Health Center, Montreal, QC, Canada H4A 3J1; ^5^Oral Biological Medicinal Science, University of British Columbia, Vancouver, BC, Canada V6T 1Z3

## Abstract

*Aim*. In routine histopathology, decalcification is an essential step for mineralized tissues. The purpose of this study is to evaluate the effects of different decalcification solutions on the morphological and antigenicity preservation in Sprague Dawley (SD) rat femurs.* Materials and Methods*. Four different decalcification solutions were employed to remove the mineral substances from rat femurs, including 10% neutral buffered EDTA, 3% nitric acid, 5% nitric acid, and 8% hydrochloric acid/formic acid. Shaking and low temperature were used to process the samples. The stainings of hematoxylin-eosin (HE) and immunohistochemical (IHC) were employed to evaluate the bone morphology and antigenicity.* Key Findings*. Different decalcification solutions may affect the quality of morphology and the staining of paraffin-embedded sections in pathological examinations. Among four decalcifying solutions, 3% nitric acid is the best decalcifying agent for HE staining. 10% neutral buffered EDTA and 5% nitric acid are the preferred decalcifying agents for IHC staining.* Significance*. The current study investigated the effects of different decalcifying agents on the preservation of the bone structure and antigenicity, which will help to develop suitable protocols for the analyses of the bony tissue.

## 1. Introduction

Decalcification is an essential step routinely performed for histopathological observation of bone and bone-containing tissues [1]. Problems arise during tissue sectioning and processing because of the mineral content in a densely packed organic extracellular matrix structure consisting of both collagenous and noncollagenous materials [2]. Minerals, mainly in form of calcium and phosphorus insoluble salts called hydroxyapatite (HA), account for sixty-five percent of bone tissue [3, 4]. HA crystals bound to the organic protein matrix provide the bone hardness and are the cause of the resistance during tissue cutting using regular microtomes [5]. In addition, the current decalcifying methods are characterized by laborious procedures and the frequent loss of immunoreactivity [1], which may impede genuine understanding of bone remodeling in the development of skeletal diseases. Therefore, the development of an efficient decalcifying method is an ongoing challenge for a high-quality processing of paraffinized bone samples.

Efficient decalcification protocols will allow the removal of insolvable inorganic salts from bony tissues, which will soften bone and teeth for easy sectioning [6]. Currently, there are several decalcification solutions available which include inorganic and organic acids, a neutral fluid containing a chelating agent, or a mixture of solutions [7–9]. An ideal decalcifying approach is to preserve the tissue morphology and antigenicity [9]. Low processing temperature, low acidity of the decalcification solution, and continuous sample shaking contribute to an efficient decalcifying and preserving the tissue structure and antigenicity of the samples [10–12].

Hematoxylin-eosin (HE) staining is one of the principal stains in histopathology and the most widely used stain in medical diagnosis. Immunohistochemistry refers to the process of detecting antigens (e.g., proteins) in cells of a tissue section by exploiting the principle of antibodies binding specifically to antigens in biological tissues to realize the qualitative and quantitative analysis of antigen [13]. Insulin-like growth factor 1 (IGF-1), the most abundant growth factor in bone, has the endocrine and paracrine actions during the process of bone remodeling [14]. In addition, IGF-1 positively correlates with bone mineral density in osteoporosis and is important for the investigation of bone formation [15].

In order to find the most suitable decalcifying agent, we evaluated four different decalcifying solutions for the whole femurs of adult rats by evaluating the time of decalcification, ease of tissue slicing, morphological preservation with HE staining, and the preservation of antigenicity for bone proteins such as IGF-1 using immunohistochemical (IHC) staining.

## 2. Materials and Methods

### 2.1. Chemicals and Antibody

All chemical reagents were purchased from Beijing Sinopharm Chemical Reagents Co. Ltd. (Beijing, China). Rabbit polyclonal IGF-1 antibody was from Santa Cruz Biotechnology, Inc. (Cat#: sc-9013. Texas, USA).

### 2.2. Animal Feeding

Six 16-week-old female Sprague Dawley (SD) rats (300 ± 10 g) were purchased from China Huafukang Animal Technology Co. Ltd. (License number: SCXK (Beijing) 2011-0004, Beijing, China). Animals were housed at clean level conditions (certification number SCXK (Jing) 2011-0024) at Beijing University of Chinese Medicine (BUCM) with the temperature of 22 ± 1°C, humidity of 55 ± 5%, and a 12 h light/dark cycle. All rats were allowed free access to tap water and food. The study protocol was approved by the animal care committee of BUCM, China.

After one week of acclimation, the rats were anesthetized with pentobarbital sodium (1% sodium pentobarbital, 0.4 mL/100 g, i.p.). The bilateral femurs were harvested and stored at −80°C for further analyses.

### 2.3. Decalcification

The femurs were prefixed in 4% paraformaldehyde for 24 h. Samples were then rinsed in running tap water for 24 h and incubated with four different decalcifying solutions (Table 1): (1) 10% EDTA (pH 7.4); (2) 3% nitric acid; (3) 5% nitric acid; and (4) 8% hydrochloric acid/formic acid followed by neutralizing with 0.1% aqueous ammonia solution for 30 min. Decalcification was performed at 4°C under continuous shaking. The decalcifying solutions were changed on a daily basis and the total decalcification time was recorded.

The decalcification process was ended when the bone was easily penetrated through by a needle without any force. Subsequently, samples were washed in running tap water for 24 h and then followed by routine dehydration and paraffin embedding. 5 *μ*m sections were cut using a Leica microtome (Leica, Germany) and placed on adhesive-precoated (5% poly-L-lysine) glass slides.

The quality of decalcification was evaluated by the following criteria: (1) the time of decalcification; (2) the ease of sectioning; (3) the morphological preservation by HE staining; (4) the antigenicity preservation by IHC staining. The ease of sectioning and morphological preservation were graded from 1 to 4 (1: poor, 2: fair, 3: good, and 4: excellent) [16].

### 2.4. HE Staining

HE staining was conducted according to routine protocols [17]. Briefly, after deparaffinization and rehydration, 5 *μ*m longitudinal sections were stained with hematoxylin solution for 5 min followed by 5 dips in 1% acid ethanol (1% HCl in 70% ethanol) and then rinsed in distilled water. Then the sections were stained with eosin solution for 3 min and followed by dehydration with graded alcohol and clearing in xylene. The mounted slides were then examined and photographed using an Olympus BX53 fluorescence microscope (Tokyo, Japan). The staining intensity of the trabecular bone was analyzed by Image-Pro Plus 6.0 software and expressed as IOD value.

### 2.5. Immunohistochemical Staining

5 *μ*m longitudinal sections of the paraffin-embedded femurs were kept at 60°C for 24 h in the oven and then followed by deparaffinizing with xylene and hydrating with an ethanol gradient (100%–70%). After successively incubating with antigen retrieval solution (Shanghai Shunbai Biotechnology Company; Shanghai, China) and 3% H_2_O_2_ for 30 min, the slides were rinsed with water and incubated with the primary antibody (IGF-1 (1 : 50)) overnight at 4°C. For negative controls, the primary antibody was replaced by nonimmunized serum. The next day, the slides were rinsed and incubated with the corresponding secondary antibody (Beijing Biosynthesis Biotechnology Co. Ltd.; Beijing, China) for 30 min followed by 3,3′-diaminobenzidine (DAB) and hematoxylin staining, respectively. The slides were then examined and photographed using an Olympus BX53 fluorescence microscope (Tokyo, Japan). The DAB staining was analyzed by Image-Pro Plus 6.0 software.

### 2.6. Statistics Analysis

One-way ANOVA was used to evaluate the effect of decalcifying solutions in the quantitative analyses of HE staining and IGF-1 antigenicity preservation. Six sections from different rats were taken for histological analyses. Student's *t*-test was used to analyze the difference between groups. A value of *p* < 0.05 was considered significant and *p* < 0.01 considered statistically significant.

## 3. Results

### 3.1. Time of Decalcification

Decalcification in 10% EDTA (pH 7.4) required 21 days. The other decalcifying solutions needed 8 days for decalcification with no obvious differences in the decalcification time.

### 3.2. Ease of Sectioning

**Figure 1:**
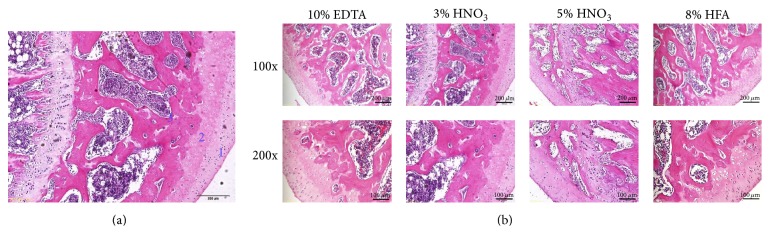
Representative microstructure ((a); ×100) and histological images (b) of HE staining showed the influences of different decalcified solutions on morphological structure preservation of rat femurs. 1 to 3 represent superficial, cartilage, and subchondral zone, respectively. HFA represents hydrochloric acid/formic acid.

The microstructure of rat femurs (HE staining) is shown in Figure 1(a), including superficial zone, calcified cartilage zone, and subchondral bone. The sections will be easily cut if bony tissues are well decalcified. The quality of morphological preservation in the section is evaluated from trabecular bone staining, cartilage tissue staining, and contrast ratio. As shown in Table 2 and Figure 1(b), different decalcification solutions resulted in differences in the easiness of tissue cutting and morphological preservation. The following order for cutting easiness was observed: 8% hydrochloric acid/formic acid = 3% nitric acid > 5% nitric acid = 10% EDTA (pH 7.4). In terms of the quality of morphological structure preservation, 3% nitric acid was the best, followed by 5% nitric acid, and then 10% EDTA (pH 7.4) and 8% hydrochloric acid/formic acid subsequently.

### 3.3. Morphological Preservation

**Figure 2:**
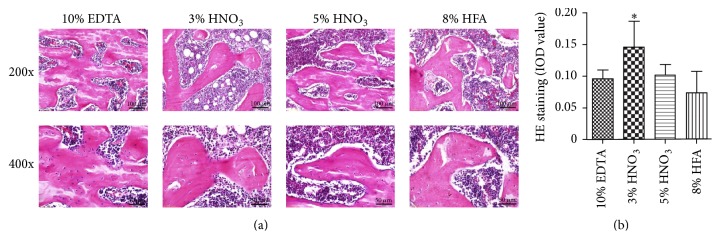
Representative images (a) and histological images analysis (b) of HE staining showed the influences of different decalcified solutions on staining quality of rat femurs. Image-Pro Plus was used to quantify the relative IOD value of HE staining of the trabecular bone. ^*∗*^*p* < 0.05 compared with EDTA group.

As shown in Figures 1(b), 2(a), and 2(b), HE staining demonstrated that different decalcifying solutions affected the staining intensity of the sections. The methods including 10% EDTA (pH 7.4), 5% nitric acid, and 8% hydrochloric acid/formic acid had very similar effects on the staining intensity of slides. However, 3% nitric acid resulted in the best brightness and uniformity of staining. And the sections processed by 8% hydrochloric acid/formic acid showed uneven staining.

### 3.4. Antigenicity Preservation

**Figure 3:**
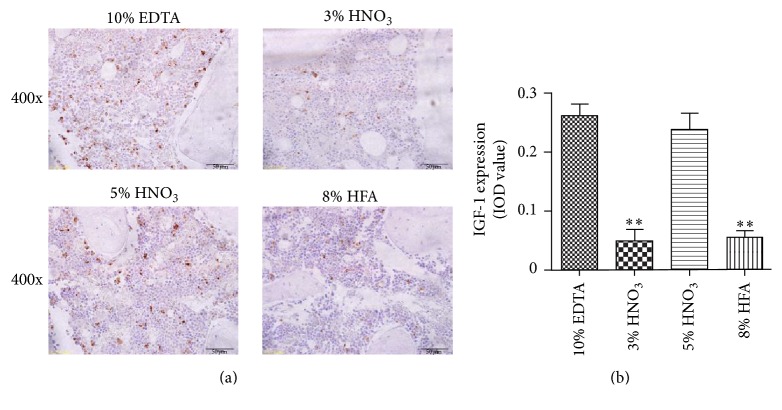
Representative immunohistochemical images (a) and histological images analysis (b) of IGF-1 expression in rat femurs showed the influences of different decalcified solutions on antigenicity preservation. Image-Pro Plus was used to analyze the relative IOD value of IGF-1. ^*∗∗*^*p* < 0.01 compared with EDTA group.

The preservation of antigen was evaluated by IHC staining. As shown in Figure 3, the positive signals (brown particles) were best observed after decalcifying with 5% nitric acid and 10% EDTA (pH 7.4). The results indicate that the decalcification solution of 10% EDTA (pH 7.4) and 5% nitric acid had a better capacity in retaining antigen preservation of IGF-1 in comparison with that of 3% nitric acid and 8% hydrochloric acid/formic acid.

## 4. Discussion

In the present study, we compared the outcome of four different decalcifying solutions using SD rat femurs on the quality of HE and IHC staining. With regard to the HE staining and morphology preservation, 3% nitric acid gives better results than 10% EDTA (pH 7.4), 5% nitric acid, and 8% hydrochloric acid/formic acid. In the case of preserving the antigenicity of the tissue samples, 10% EDTA (pH 7.4) is found to be the most optimal solution followed by 5% nitric acid, 3% nitric acid, and 8% hydrochloric acid/formic acid.

EDTA is one of the most commonly used decalcifying agents. It preserves well antigenicity as shown in this study. However, the advantage of EDTA as a decalcifying agent in a routine setting is masked by time-consuming incubation, especially for large-sized samples [1, 18]. Decalcification time reported for EDTA ranges from 2 to 4 months [17]. In addition, most of the investigators used EDTA for decalcification at room temperature, which may damage the antigen availability of the samples. In the current study, two variations of the standard protocols were utilized to improve bone decalcification quality: (1) continuous shaking of the samples and (2) decalcification at 4°C. The improvements appeared to allow for a better and even infiltration of the decalcifying solutions into the tissue.

An effective decalcifying method permits the generation of morphologically high-quality tissue sections. In the current study, we found that 8% hydrochloric acid/formic acid and 3% nitric acid are optimal to obtain 5 *μ*m sections of good quality. On the other hand, 3% nitric acid provides the best results regarding tissue structure preservation and contrast ratio. Ying et al. [19] found that addition of ethanol to decalcifying agent contributed to a further improvement of the HE staining quality. We also found that the addition of ethanol to mixed acids (6 mL nitric acid, 10 mL hydrochloric acid, 30 mL formic acid, 5 mL glacial acetic acid, 104 mL 70% ethanol, and 45 mL water) improved the brightness of the sections with HE staining when compared to that of 10% EDTA (data not shown). However, the addition of ethanol may damage antigenicity because no positive signal was observed during IHC staining.

The effect of different decalcifying agents on antigen preservation is obviously different. In our study, decalcifying with 5% nitric acid and 10% EDTA (pH 7.4) produced strong positive signals of IGF-1 staining. In addition, Athanasou et al. demonstrated that prolonged decalcification in strong acid may diminish the antigen activity, but the weaker acids may nevertheless better preserve the antigenic reactivity, morphology, and staining quality, allowing for time consuming for decalcifying [20]. However, prolonged decalcification may also adversely affect the staining quality [21]. Therefore, an accurate control of the decalcification duration improves the quality of sectioning and staining.

Sangeetha et al. reported that decalcification in 5% nitric acid resulted in yellow discoloration of the bone sections at ambient temperature [9]. Furthermore, microwave technique may accelerate the decalcification [9], but the sections are vulnerable to disintegration and falling off from the slides.

In order to improve the quality of staining [8], we also neutralized the samples after decalcification with mineral acids. Contrary to the expectation, the sections that were decalcified in 3% or 5% nitric acid were easily to fall off from the slides after neutralization. Moreover, there was no difference in respect to the quality of the staining and antigenicity preservation.

In summary, different decalcification solutions may affect the quality of the morphology and staining of paraffin-embedded bone tissue sections. Among the four methods used in this study, 3% nitric acid is the best decalcifying solution for HE staining, while 10% neutral buffered EDTA and 5% nitric acid are the best decalcifying agent for IHC staining.

## Figures and Tables

**Table 1:** The ingredients and preparation of different decalcifying agents.

	Decalcifying solution 1	Decalcifying solution 2	Decalcifying solution 3	Decalcifying solution 4
Decalcifying agents	10% EDTA	3% nitric acid	5% nitric acid	8% hydrochloric acid/formic acid
Preparation	100 g EDTA and 10 g sodium hydroxide	3 mL nitric acid	5 mL nitric acid	8 mL hydrochloric acid and 8 mL formic acid
Distilled water	Add to 1000 mL	Add to 100 mL	Add to 100 mL	Add to 100 mL
pH	7.4	—	—	—

**Table 2:** Decalcifying solutions scores as the measurement of ease of sectioning and morphological preservation.

Decalcifying solutions	Ease of sectioning	HE morphological evaluation				Total score
		TBS	CTS	CT	Total	
10% EDTA	3	3	3	3	9	12
3% nitric acid	4	4	3	4	12	16
5% nitric acid	3	3	4	3	10	13
8% HFA	4	2	4	3	7	12

Note: TBS: trabecular bone staining, CTS: cartilage tissue staining, CT: contrast ratio, HFA: hydrochloric acid/formic acid.
